# Supplementary material for: Content-rich biological network constructed by mining PubMed abstracts
Source: BMC Bioinformatics. 2004 Oct 8;5:147. doi: 10.1186/1471-2105-5-147 (PMC528731; doi:10.1186/1471-2105-5-147)
Supplement: Additional File 5 — The original Chilibot query results of the term "long-term potentiation (LTP)" and 22 other terms, limiting the latest references analyzed to the years 1990, 1995, 2000, and 2004. [file 1471-2105-5-147-S5.bz2 › chilibotAdditionalFile5/ltp2004/html/PKA_CAMKIV.html]

 


 **PKA** and **CAMKIV** 
  
Found 6 abstracts in PubMed,  **6 abstracts were retrieved and analyzed**.  


---

 Search Google  |
 PDF files only 
|  EDU domain only 

---

**Interactive relationship** (e.g. stimulation, inhibition, etc)

- The  **CaMKIV**  mediated gene induction requires the activity of CREB ATF family members but is independent of  **PKA**  activity.  Ref: 8065343 Mol Cell Biol, 1994
- In the cytoplasm, there is extensive cross talk between CaMKK,  **CaMKIV**  and other signaling cascades, including those that involve the cAMP dependent kinase  **PKA** , MAP kinases and protein kinase B PKB.  Ref: 10366852 Trends Biochem Sci, 1999

**Parallel relationship** (e.g. studied together, co-existance, homology, etc.)

- MEKK and CaMKII... increased promoter activity 4.5 and 1.5 fold, respectively, in NB2A cells.  Ref: 12421350 J Neurochem, 2002
- Inhibition of  **PKA**  or the MAPK RSK cascades were without effect on the late phase, while constructs designed to interfere with  **CaMKIV**  function attenuated the late phase.  Ref: 10433267 Neuron, 1999
- Activation is classically brought about by signaling dependent phosphorylation of a key acceptor site by a number of possible kinases, including  **PKA** ,  **CamKIV** , and Rsk 2.  Ref: 11969286 Exp Cell Res, 2002
